# MetaMiner: a tool to retrieve, normalize and visualize the genomic metadata of prokaryotes

**DOI:** 10.1093/bib/bbaf631.050

**Published:** 2025-12-12

**Authors:** Jaykumar Kiritkumar Patel, Ravikrishnan Elangovan

**Affiliations:** Department of Biochemical Engineering and Biotechnology, Indian Institute of Technology Delhi, New Delhi, 110016; Department of Biochemical Engineering and Biotechnology, Indian Institute of Technology Delhi, New Delhi, 110016

## Abstract

**Aim:**

The rapid advancement of high-throughput sequencing technologies has led to an exponential increase in microbial genome sequences in public databases. However, the utility of these genomes largely depends on the quality and consistency of their accompanying metadata. Inconsistent, erroneous, or incomplete metadata can significantly hinder the integration and reuse of genomes in broader genomic analyses.^1^ While manual standardization of metadata is possible, it becomes increasingly cumbersome as genome numbers grow.^2,3^ To address this challenge, we introduce MetaMiner, a GUI tool designed to retrieve, normalize, and standardize metadata efficiently.

**Methods:**

Preliminary evaluation of MetaMiner involved testing its ability to retrieve and standardize metadata for *Acinetobacter baumannii* genomes. Its performance was compared to existing NCBI retrieval tools and manual metadata standardization. To facilitate intuitive exploration, processed metadata was further integrated into an interactive dashboard.

**Results:**

Results revealed better performance in data retrieval and transformation, while data normalization was nearly comparable to manual curation. Beyond cleaning, tool integrates processed data into an interactive dashboard, offering comprehensive visualizations of genome assembly statistics, biosample attributes, submission trends, and annotations. Users can apply custom filters and sorting options, facilitating intuitive exploration and analysis of large genomic datasets.

**Conclusion:**

By automating metadata curation and providing an accessible overview of genomic resources, MetaMiner enhances both accessibility and reusability of publicly available genomes.

**References:**

1. Caliskan A. et al. ‘Metadata integrity in bioinformatics: bridging the gap between data and knowledge.’ Computational and Structural Biotechnology Journal 2023;21:4895–4913.

2. Musen M.A. et al. ‘Modeling community standards for metadata as templates makes data FAIR.’ Scientific Data 2022;9.

3. Batista D. et al. ‘Machine actionable metadata models.’ Scientific Data 2022;9.

